# ROOT HAIR DEFECTIVE SIX-LIKE Class I Genes Promote Root Hair Development in the Grass *Brachypodium distachyon*

**DOI:** 10.1371/journal.pgen.1006211

**Published:** 2016-08-05

**Authors:** Chul Min Kim, Liam Dolan

**Affiliations:** 1 Department of Plant Sciences, University of Oxford, Oxford, United Kingdom; 2 Oxford Martin School, University of Oxford, Oxford, United Kingdom; Peking University, CHINA

## Abstract

Genes encoding ROOT HAIR DEFECTIVE SIX-LIKE (RSL) class I basic helix loop helix proteins are expressed in future root hair cells of the *Arabidopsis thaliana* root meristem where they positively regulate root hair cell development. Here we show that there are three RSL class I protein coding genes in the *Brachypodium distachyon* genome, *BdRSL1*, *BdRSL2* and *BdRSL3*, and each is expressed in developing root hair cells after the asymmetric cell division that forms root hair cells and hairless epidermal cells. Expression of BdRSL class I genes is sufficient for root hair cell development: ectopic overexpression of any of the three RSL class I genes induces the development of root hairs in every cell of the root epidermis. Expression of BdRSL class I genes in root hairless *Arabidopsis thaliana root hair defective* 6 (*Atrhd6) Atrsl1* double mutants, devoid of RSL class I function, restores root hair development indicating that the function of these proteins has been conserved. However, neither AtRSL nor BdRSL class I genes is sufficient for root hair development in *A*. *thaliana*. These data demonstrate that the spatial pattern of class I RSL activity can account for the pattern of root hair cell differentiation in *B*. *distachyon*. However, the spatial pattern of class I RSL activity cannot account for the spatial pattern of root hair cells in *A*. *thaliana*. Taken together these data indicate that that the functions of RSL class I proteins have been conserved among most angiosperms—monocots and eudicots—despite the dramatically different patterns of root hair cell development.

## Author Summary

Root hairs are tubular extensions that extend from specialized cells in the root surface. They take up nutrients and water from the soil and tether the root to its substrate. The differentiation of root hair cells in the cress family is controlled by a group of regulators called RSL class I transcription factors. The spatial arrangement of root hair cells in grasses is very different from cresses like *Arabidopsis thaliana*. Root hair cells form in discrete longitudinal files in cresses: there are stripes of root hair cells that alternate with stripes of hairless epidermal cells. Root hair cells alternate with hairless epidermal cells in a chessboard pattern in the root epidermis of grasses. We show that the pattern of RSL class I gene expression defines the pattern of root hair cell differentiation in the root epidermis of the grass *Brachypodium distachyon* but not in the cress *Arabidopsis thaliana*; ectopic expression of RSL genes can transform every cell into a root hair cell in the grass but not in the cress. Despite these differences in development we also show that the function of RSL class I genes has been conserved since these genes last shared a common ancestor approximately 200 million years ago.

## Introduction

Root hairs are filamentous extensions of epidermal cells that extend the absorbing surface of roots into the surrounding soil. They play essential functions in nutrient acquisition and are particularly important for the uptake of nutrients with limited soil mobility such as phosphate [1,2]. The spatial pattern of root hair cell and hairless epidermal cell differentiation varies among angiosperms [3–6]. In many taxa—including the grass family, the Poaceae—root hair cells alternate with hairless epidermal cells along every epidermal cell file [4,7]. In other taxa—including the cress family, the Brassicaeae—cell files comprising only root hair cells are flanked by two or more files that contain only hairless epidermal cells [8]. In Brassiceae files of root hair cells are located between a pair of underling cortical cells while hairless epidermal cell files develop over single cortical cells [9,10]. In the Poaceae the two epidermal cell types develop in any position relative to underlying cortical cells [4,7].

The differentiation of root hair cells in *A*. *thaliana* is positively regulated by the activity of ROOT HAIR DEFECTIVE SIX-LIKE (RSL) class I basic helix transcription factors in future root hair cells [11]. The striped pattern of epidermal cell types that develops results, in part, from RSL class I transcription and translation in cells overlying longitudinal cortical cell junctions and the transcriptional repression of these genes in the epidermal cells overlying cortical anticlinal walls. The transcriptional repressor GLABRA2 accumulates in the future non-hair cells and represses RSL transcription; class I RSL genes are expressed in root epidermal cells in which *GL2* is not expressed [12]. The spatial pattern of *GL2* expression is determined by a signaling system, which produces a transcriptionally active complex—containing the WEREWOLF (WER) Myb transcriptional activator—in the future hairless epidermal cell files that promotes *GL2* expression and an inactive complex (containing the CAPRICE Myb transcriptional repressor) in the future hair cell files [13–15].

*A*. *thaliana* RSL class I genes are expressed in future root hair cells located in the meristem [11]. The expression of RSL class I in the future hair cells positively regulate the expression of RSL class II genes in the elongation zone and these genes promote root hair initiation and elongation. A key RSL class II gene is *AtRSL4*, which is sufficient for root hair elongation; loss of *AtRSL4* function results in the development of fewer and shorter root hairs while constitutive expression results in the constitutive elongation of root hair cells [16].

The grass (Poaceae) root epidermis comprises files of cells in which hair cells alternate with hairless epidermal cells. In *Brachypodium distachyon* this alternating pattern is the result of asymmetric mitoses which form smaller daughters cell that differentiate as a root hair cells, and larger cells that differentiate as hairless epidermal cells [7,17,18]. Genetic analysis has identified one transcriptional regulator of root hair cell development in *O*. *sativa*. *ROOT HAIRLESS1* (*OsRHL1)* encodes a group XI basic helix loop helix transcription factor that is required for root hair cell development. Plants homozygous for loss of function *Osrhl1-1* mutations initiate root hairs but they do not elongate [19]. Closely related homologs positively regulate root hair development in *Lotus japonicus* and *A*. *thaliana* [20,21]. It is likely that *OsRHL1* promotes the expression of genes required for the growth or root hairs. *B*. *distachyon TRYPTOPHAN AMINOTRANSFERASE OF ARABIDOPSIS RELATED2* (*BdTAR2)* is required for auxin biosynthesis in the root and for root hair elongation; *Bdtar2* loss of function mutants develop shorter root hairs than wild type [22].

Given the central regulatory role played by RSL class I genes during root hair development *in A*. *thaliana* we tested the hypothesis that *RSL* genes positively regulate root hair development in the grass *B*. *distachyon*. We show here that class I RSL genes promote root hair development and expression is sufficient for root hair development. This suggests that the function of RSL genes in promoting root hair cell differentiation is conserved among monocots and eudicots,

## Results

### RSL class I genes are present in grass and cereal genomes

**Fig 1:**
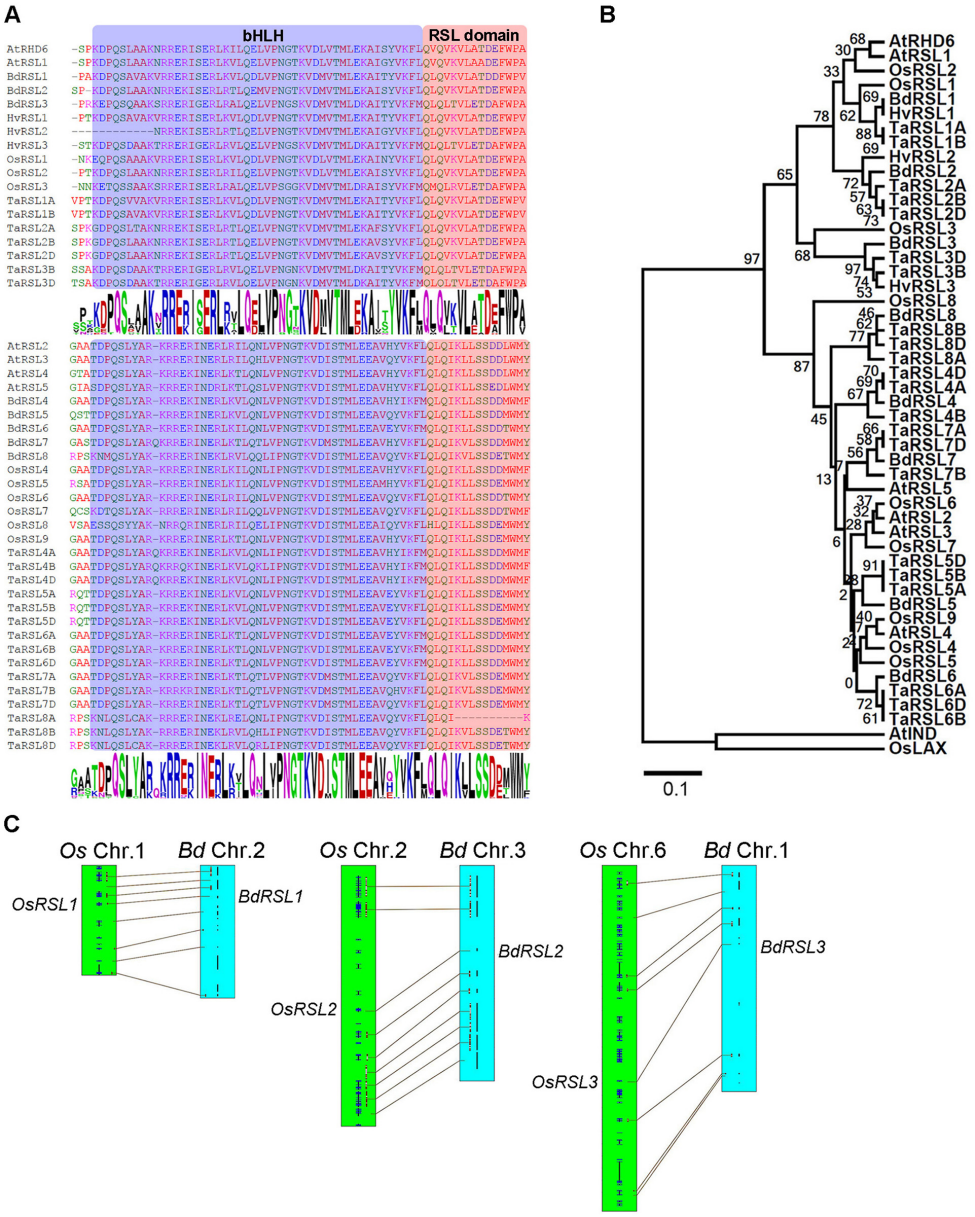
Genes encoding RSL class I proteins are present in the genomes of members of the grass family. **(A)** Alignment of conserved regions of *Brachypodium distachyon* (Bd), *Oryza sativa* (Os), *Triticum aestivum* (Ta), *Hordeum vulgare* (Hv) and *Arabidopsis thaliana* (At) RSL class I proteins. The position of the bHLH and RSL domains is indicated by coloured boxes shaded blue and red respectively. The sequence logos represent the multiple alignment of RSL class I and class II amino acid sequences from five plant species (S1A Fig); heights are proportional to amino acid conservation in each position. **(B)** Maximum-likelihood tree showing the relationship between RSL class I and class II proteins from *Brachypodium distachyon* (Bd), *Oryza sativa* (Os), *Triticum aestivum* (Ta), *Hordeum vulgare* (Hv) and *Arabidopsis thaliana* (At) based on the protein sequence of bHLH and RSL domains. AtbHLH040 (AtIND) and OsbHLH123 (OsLAX) were used as out groups. Numbers below branches indicate bootstrap percentages. **(C)** Synteny between RSL class I genes of *B*. *distachyon* and *O*. *sativa*. Connecting lines between linkage groups define chromosome regions with collinear orthologous genes. The locations of *RSL1*, *RSL2* and *RSL3* orthlogs are indicated.

To determine if RSL class I genes control the development of root hair cells in *B*. *distachyon* we searched for similar genes in the genomes of members of the grass family (Poaceae) (S1A Fig). We discovered genes encoding proteins with the conserved RSL domain next to the bHLH domain (Fig 1A). The topology of gene trees constructed using alignments of the basic helix-loop-helix domain and conserved RSL motif from these proteins showed that three *B*. *distachyon* genes (*BdRSL1*, *BdRSL2* and *BdRSL3*) (Fig 1B and S1B Fig) and three *O*. *sativa* genes–(*OsRSL1*, *OsRSL2* and *OsRSL3*) were most closely related to the previously characterized RSL class I genes *AtRHD6* and *AtRSL1* of *A*. *thaliana*. BdRSL1, BdRSL2 and BdRSL3 are respectively 84%, 89% and 73% identical to AtRHD6 in the bHLH-RSL domain (S1 Table) but there is no conservation outside these conserved regions. Gene order (synteny) indicates that *OsRSL1* and *BdRSL1*, *OsRSL2* and *BdRSL2*, and *OsRSL3* and *BdRSL3* are orthologous gene pairs (Fig 1C). RSL class I genes were also identified in genomes of other members of the grass family including *Hordeum vulgare* and *Triticum aestivum*. The RSL class I clade was sister to a clade that contained the *A*. *thaliana* RSL class II proteins, AtRSL2, AtRSl3, AtRSL4 and AtRSL5. Five *B*. *distachyon* proteins, six *O*. *sativa* and 15 *T*. *aestivum* proteins were identified that belonged to the RSL class II clade. Taken together these data indicate that RSL class I and RSL class II genes are present in the genomes of members of the grass family.

### RSL class I genes are expressed in developing epidermal cells of *Brachypodium distachyon*

**Fig 2:**
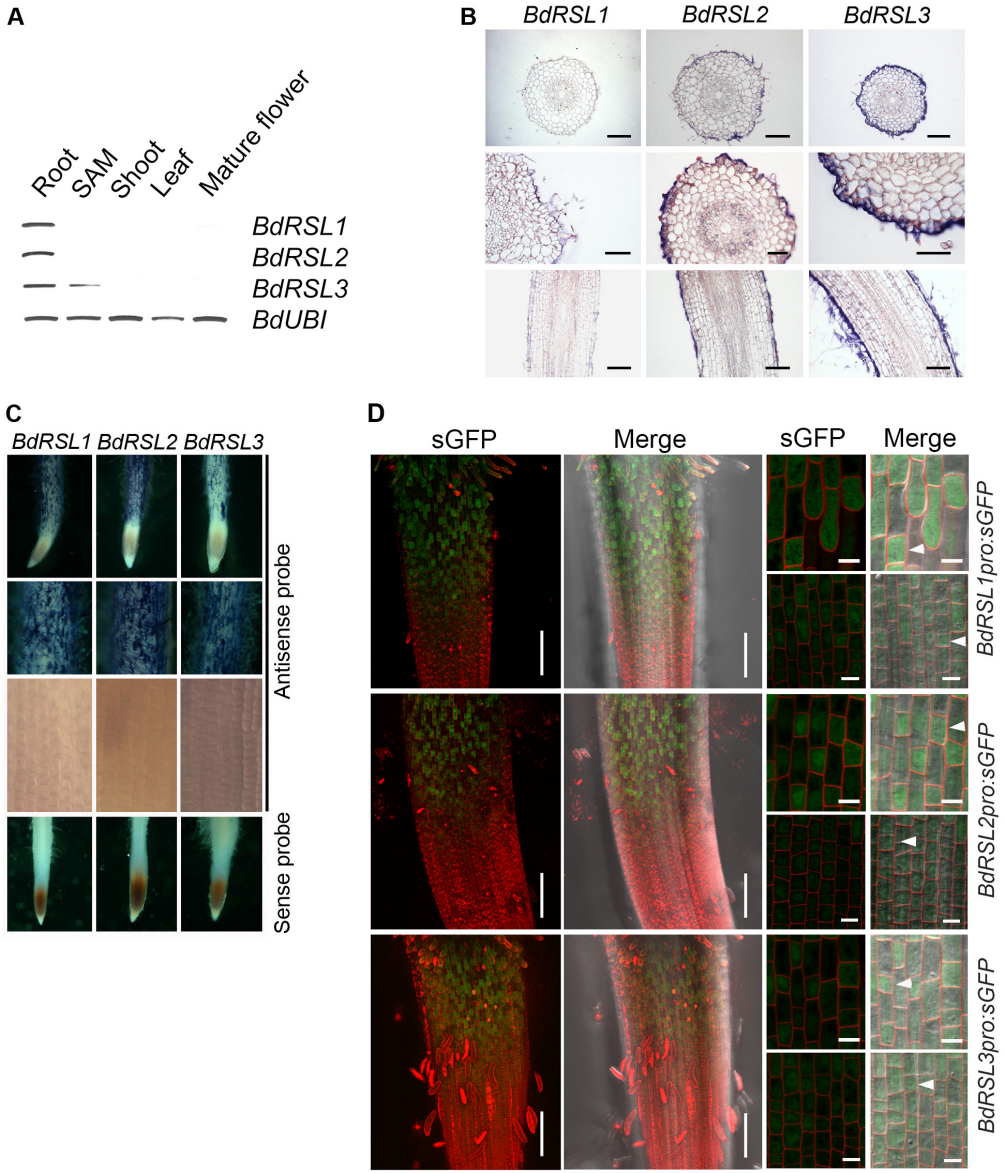
RSL class I genes are expressed in *B*. *distachyon* root hair cells. **(A)** Representative semi-quantitative RT-PCR analysis of steady state levels of RSL class I mRNA in different tissues and organs; root, shoot apical meristem (SAM), shoot, leaf and mature flower. **(B)** Gene specific probes were hybridized to transverse sections (top and middle) and longitudinal sections of roots (bottom) in the regions where root hairs are initiated. Hybridization of the probe is restricted to the epidermis. Scale bars 100 μm. **(C)** Whole mount in situ hybridization showing that RSL class I mRNAs accumulate in developing root hair cells. The top three rows were hybridized with an antisense probe; the top row shows root tips where blue precipitate indicates the presence of mRNA; the second row shows the zone where root hairs are growing and there is mRNA hybridization; the third row is the meristem where there is no mRNA hybridization. A sense probe was used as a negative control in the bottom row. **(D)** GFP fluorescence reporting the activities of *B*. *distachyon* RSL class I gene promoters. Two left-hand columns: root tips and white arrows highlight appearance of GFP fluorescence. Two right-hand columns: there are four images for each reporter construct. The top row is a representative pair of images of the root epidermis at the root hair initiation stage. White arrowheads indicate GFP fluorescence in the short cells bearing from which root hairs develop. The lower pair of images show the root epidermis at the transition between meristem and elongation zone where the asymmetric cell division has occurred. White arrowheads indicate GFP fluorescence in a smaller daughter cell resulting from an asymmetric cell division. Scale bars 50 μm (two left-hand columns), 10 μm (two right-hand columns).

RSL class I genes are expressed in the *A*. *thaliana* root meristem and mRNA disappears from cells before root hair initiation. We set out to determine if this expression pattern is conserved in *B*. *distachyon*. First, using RT-PCR we detected *BdRSL1*, *BdRSL2* mRNA only in roots while *BdRSL3* mRNA transcript was present in the roots and in the shoot apical meristem (Fig 2A). Second, to identify the cells of the root where RSL class I mRNA accumulated, we carried out in situ hybridization experiments on sectioned and whole mount roots. Hybridization of gene-specific probes to sections of roots revealed that *BdRSL1*, *BdRSL2* and *BdRSL3* mRNA accumulated in epidermis and not in any other tissues of the root (Fig 2B). Third, whole mount in situ hybridization showed that RSL class I RNA transcripts were not detected in the meristem (Fig 2C). *BdRSL1*, *BdRSL2* and *BdRSL3* mRNA was first detected at the border between the meristem and elongation zone. mRNA was detectable in these cells as they expanded in the elongation zone, and initiated root hairs and elongated root hairs in the differentiation zone (Fig 2C). These data suggest that RSL class I genes are expressed post-mitotically in developing root hair cells and continue to be expressed during root hair morphogenesis. To verify independently that RSL class I genes were expressed in developing root hair cells in the elongation and differentiation zones, we identified the cells in which the RSL class I promoters were active. We used plants transformed with gene constructs in which the RSL class I promoter controlled expression of the synthetic Green Fluorescent Protein (sGFP) [23]. GFP fluorescence was not detected in the meristem of *BdRSL1pro*:*sGFP*, *BdRSL2pro*:*sGFP or BdRSL3pro*:*sGFP* transformed plants, confirming the conclusion that these genes are not expressed in the dividing cells of the root (Fig 2D). However, GFP fluorescence was detected in developing root hair cells in the elongation zone, and in the differentiation zone where the root hairs actively elongated (Fig 2D and S2 Fig). The earliest detectable fluorescence was found in cells at the beginning of the elongation zone (after the completion of asymmetric mitosis). While the promoters of each of the three RSL class I genes was preferentially expressed in the smaller daughter cells (Fig 2D) they were occasionally active in the large daughter cells. In *BdRSL1pro*:*sGFP* roots shown in Fig 2D, seven of the eight small cells in the field of view expressed GFP and none of the seven long expressed GFP (Fig 2D). In *BdRSL2pro*:*sGFP* roots all 14 of the short cells in the field of view expressed GFP while one of the 13 long cells expressed GFP (Fig 2D). In *BdRSL3pro*:*sGFP* roots all 16 of the short cells in the field of view expressed GFP while two of the 17 long cells expressed GFP. These data indicate that the RSL class I genes are preferentially expressed in the smaller daughter cell that forms from the asymmetric mitosis that forms a root hair cell and a hairless epidermal cell pair. GFP fluorescence was detected later in development in cells with elongating root hairs (S2 Fig). These data indicate that RSL class I genes are preferentially expressed in developing root hair cells, from at or just after the formative asymmetric cell division and continues through root hair elongation. This expression pattern is different from that observed in *A*. *thaliana* where RSL class I genes are expressed in the meristem and expression is not detectable in cells with growing root hairs. Despite the differences in expression pattern, these data are consistent with the hypothesis that RSL class I genes positively regulate root hair cell development in *B*. *distachyon*.

### RSL class I genes are sufficient for root hair cell development in *Brachypodium distachyon*

**Fig 3:**
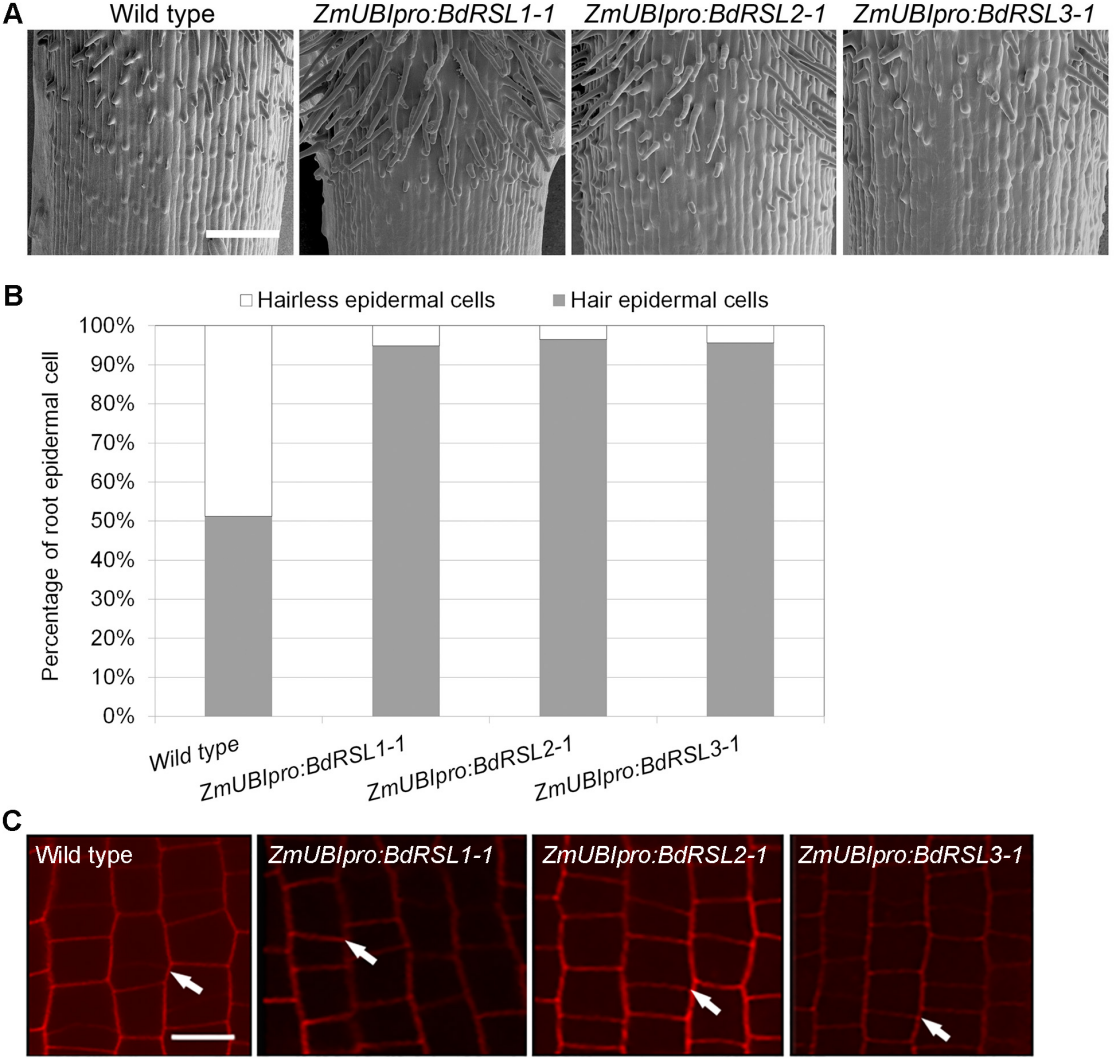
RSL class I expression is sufficient for root hair cell development in *B*. *distachyon*. **(A)** Plants transformed with *ZmUBIpro*:*BdRSL1*, *ZmUBIpro*:*BdRSL2* or *ZmUBIpro*:*BdRSL3* develop root hairs on almost every root epidermal cell. Scale bar 100 μm. **(B)** Percentage root hair cells (grey bar) and percentage hairless epidermal cells (white bar). **(C)** Asymmetric mitoses occur in plants that overexpress RSL class I genes. White arrows indicate the location of the new cell wall formed after asymmetric division forms a relatively small cell and a relatively large cell. Scale bar 10 μm.

To determine if RSL class I proteins are sufficient for root hair development in the root epidermis, the relative numbers of root hair cells and hairless epidermal cells on wild type and plants constitutively expressing *BdRSL1*, *BdRSL2* or *BdRSL3* were compared. To overexpress each RSL class I gene constitutively, *B*. *distachyon* was transformed with constructs in which each gene was placed under the control of the promoter and first intron of the *Zea mays Ubiquitin1* gene (*ZmUBI*_*pr*o_). Plants lines in which each of the RSL class I genes was overexpressed were identified (S3A Fig). Almost every cell in the root epidermis developed root hairs in each of the overexpressing lines (Fig 3A). While 51% of epidermal cells develop as root hair cells in wild type, 95%, 96% and 96% of epidermal cells developed root hairs in *ZmUBI*_*pro*_*BdRSl1*, *ZmUBI*_*pro*_*BdRSL2* and *ZmUBI*_*pro*_*BdRSL3* lines respectively (Fig 3B). Furthermore, root hairs were longer in the RSL class I overexpressing lines than in wild type controls (S3B Fig). This demonstrates not only that RSL class I proteins positively regulate the specification of epidermal cell identity in *B*. *distachyon* but also that these genes are sufficient for root hair cell development.

The expression pattern of RSL class I genes suggests that *BdRSL1*, *BdRSL2* and *BdRSL3* act in the elongation zone after the asymmetric cell division that generates root hair cells. To test if these genes act after the formative asymmetric cell division, the positioning of new transverse cell walls was determined in plants transformed with *ZmUBI*_*pro*_*BdRSL1*, *ZmUBI*_*pro*_*BdRSL2* or *ZmUBI*_*pro*_*BdRSL3* gene constructs. In wild type, the last cell division in a file is accompanied by an asymmetric mitosis forming a small daughter cell with root hair cell fate and a larger cell with hairless epidermal cell fate (Fig 3C). Asymmetric cell divisions were observed in all cell files of the RSL class I overexpressing plants and were indistinguishable from wild type; asymmetric mitoses formed a cell pair in which one cell was larger than the other (Fig 3C). In the overexpressing plants, both daughter cells developed root hairs, in contrast to wild type where only the smaller daughter cell formed a root hair. Taken together these data indicate that RSL class I proteins are sufficient for root hair cell development in the root epidermis of *B*. *distachyon* and they likely act after the asymmetric cell division that generates the root hair cell. We conclude that the spatial pattern of root hair cell differentiation in the *B*. *distachyon* root epidermis is largely dictated by the pattern of RSL class I gene expression.

### RSL class I genes positively regulate root hair growth and likely act after the asymmetric cell division that forms root hair cells

**Fig 4:**
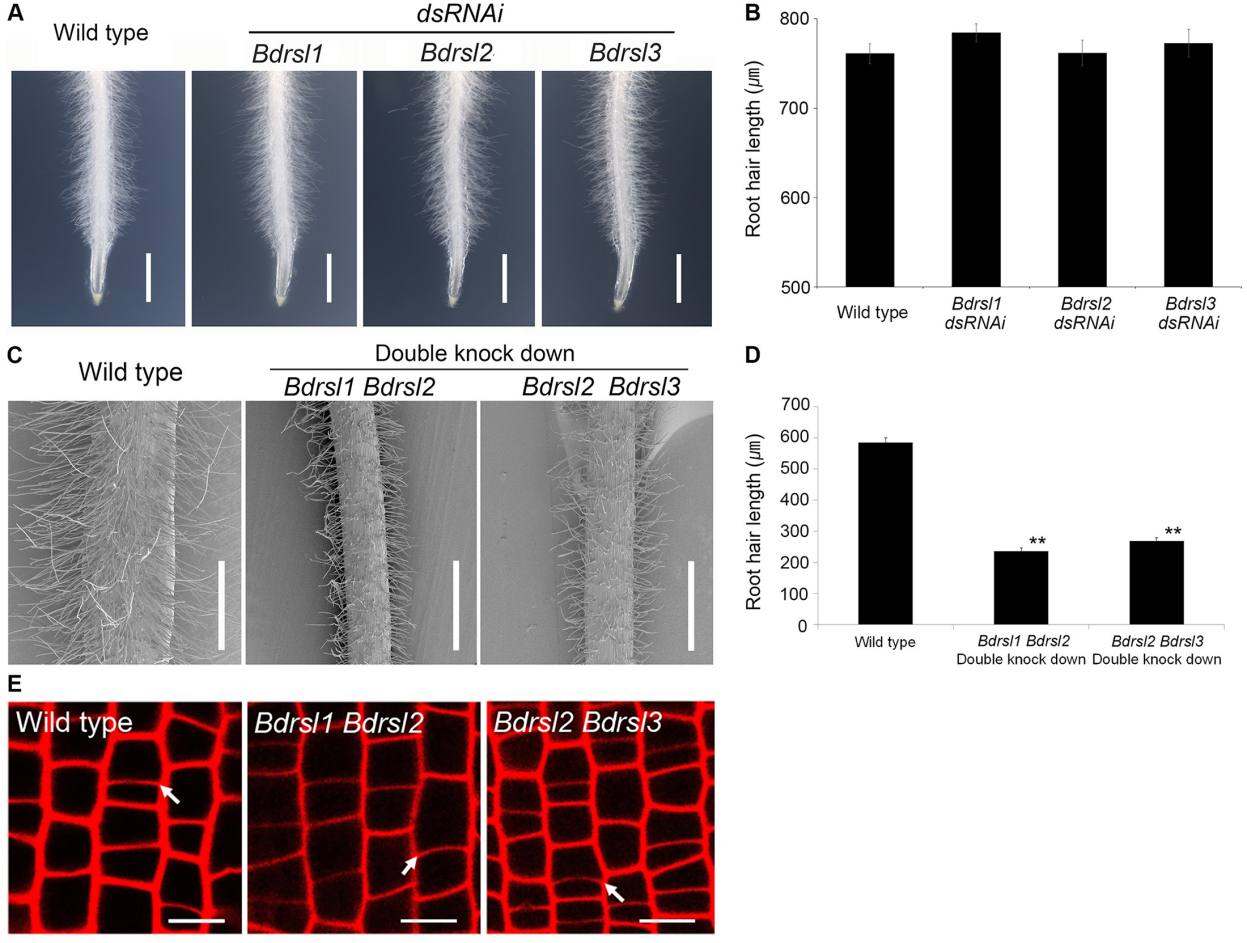
RSL class I genes act after the asymmetric cell division that forms root hair cells in *B*. *distachyon*. **(A, B)** Root hair length in *Bdrsl1*, *Bdrsl2*, and *Bdrsl3* dsRNAi single knock down lines is indistinguishable from wild type. Scale bar 10 mm. Error bar is SD. **(C, D)** Root hairs are shorter in *Bdrsl1 Bdrsl2* and *Bdrsl2 Bdrsl3* double knock down lines than that of wild type. Scale bar 500 μm. Asterisks in **(D)** indicate statistically significant differences between knock down lines and wild type at **P<0.01 (t-test). Error bar indicates SD. **(E)** Asymmetric mitoses occur in all cell files of the RSL class I double knock down plants. White arrows indicate positions of new transverse cell walls formed after asymmetric mitosis. Scale bar 10 μm.

To verify independently when RSL genes act during root hair cell development we tested if the asymmetric formative cell division occurred in transgenic lines with reduced RSL class I function. Reduced expression of individual *BdRSL1*, *BdRSL2* and *BdRSL3* genes had no impact on root hair cell development (Fig 4A and 4B), Therefore, lines with decreased expression of two RSL class I genes were constructed. Steady state levels of *BdRSL1* and *BdRSL3* mRNA were reduced by more than 50% in one set of lines while steady state levels of *BdRSL2* and *BdRSL3* mRNA were reduced by more 50% in the other set of lines (S4 Fig). Imaging of the cross walls on propidium iodide stained roots revealed that the asymmetric cell divisions occurred in *BdRSL1 BdRSL2* and *BdRSL2 BdRSL3* knock down plants and were indistinguishable from wild type (Fig 4E). These data are consistent with the hypothesis that RSL gene function does not regulate asymmetric cell division, although we cannot rule out the possibility that there may be some residual RSL1 class I activity in the double knockdown plants that is sufficient for division asymmetry. Furthermore, root hairs of the *BdRSL1 BdRSL2* double knockdown plants were 50% shorter than wild type, and root hairs on *BdRSL2 BdRSL3 double* knockdown plants were 54.0% shorter than wild type (Fig 4C and 4D). This indicates that the expression of RSL class I genes promotes root hair cell morphogenesis and differentiation. Taken together these data suggest that RSL genes positively regulate root hair development and act after the asymmetric cell division that forms hair cells in *B*. *distachyon*.

### The function of RSL class I proteins is conserved between *B*. *distachyon* and *A*. *thaliana*

**Fig 5:**
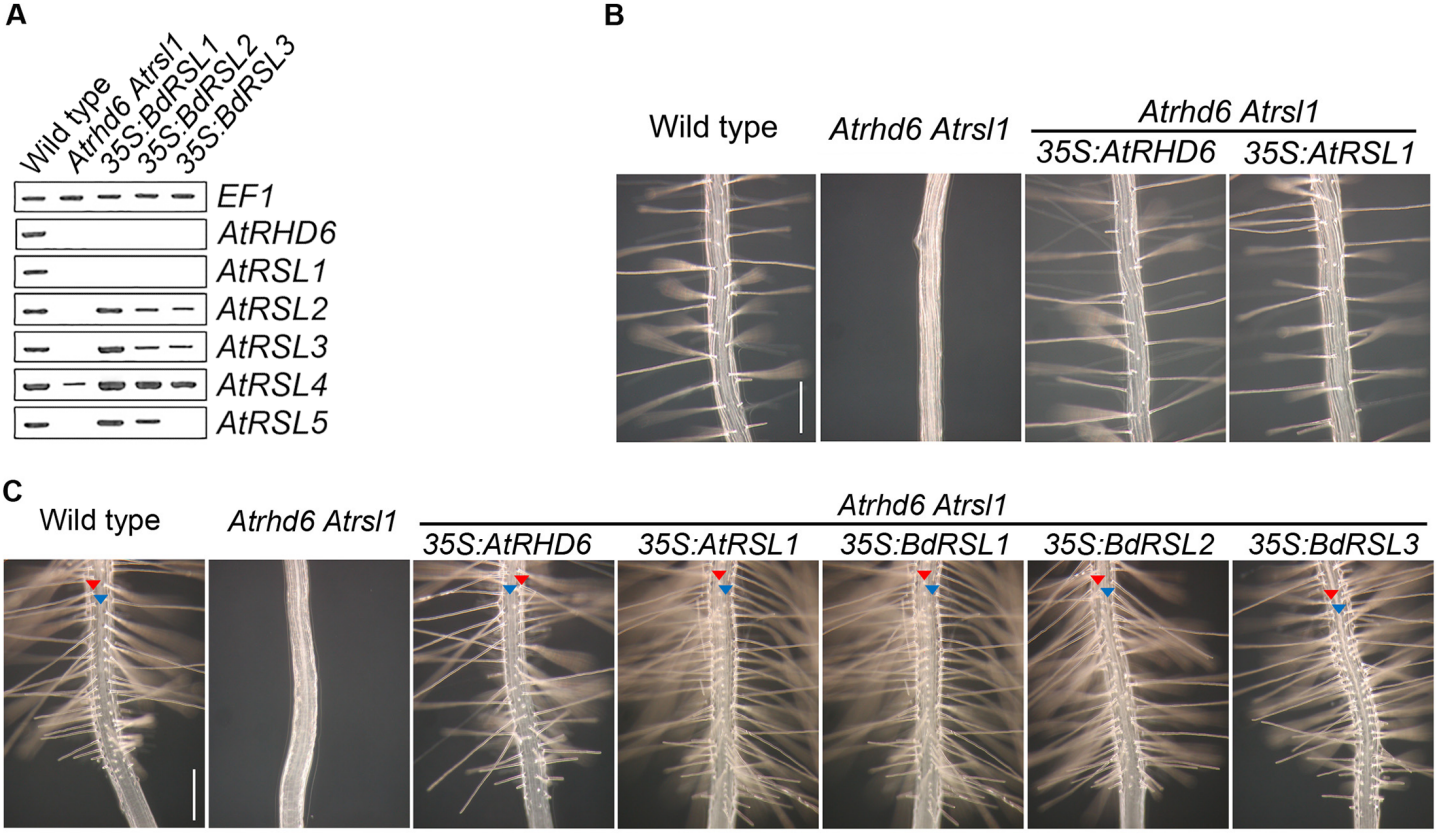
The function *B*. *distachyon* and *A*. *thaliana* RSL class I genes is conserved. **(A)** Steady state levels of *AtRSL* class I and class II mRNA in *Atrhd6 Atrsl1* double mutants, and in *Atrhd6 Atrsl1* double mutants transformed BdRSL class I genes under the control of the *35S* promoter. Lane 1: wild type, Lane2: *Atrhd6 Atrsl1* double mutant, Lane3: *Atrhd6 Atrsl1 35S*:*BdRSL1*, Lane4: *Atrhd6 Atrsl1 35S*:*BdRSL2*, Lane5: *Atrhd6 Atrsl1 35S*:*BdRSL3*. **(B) P**henotype of wild type; *Atrhd6 Atrsl1* double mutant; *Atrhd6 Atrsl1 35S*:*AtRHD6*; *Atrhd6 Atrsl1 35S*:*AtRSL1*. Scale bar 200 μm. **(C)** Files of root hair cells and hairless epidermal cells form in wild-type, *Atrhd6 Atrsl1 35S*:*AtRHD6*, *Atrhd6 Atrsl1 35S*:*AtRSL1*, *Atrhd6 Atrsl1 35S*:*BdRSL1*, *Atrhd6 Atrsl1 35S*:*BdRSL2* and *Atrhd6 Atrsl1 35S*:*BdRSL3*. *Atrhd6 Atrsl1* double mutants do not develop root hairs. Red arrowhead indicates a hair cell file, blue arrowhead indicates the position of a hairless epidermal cell file. Scale bar 200 μm.

Given the conservation of the bHLH and RSL domains of RSL class I proteins between B. *distachyon* and *A*. *thaliana*, we predicted that their function would be at least partially conserved (S1 Table). If the function of RSL class I genes was conserved since *B*. *distachyon* and *A*. *thaliana* last shared a common ancestor, we predicted that expression of RSL class I genes from *B*. *distachyon* would restore root hair cell development in root hairless *Atrhd6 Atrsl1* double mutants by re-establishing RSL class II gene expression. RSL class I genes positively regulate root hair cell development by promoting the expression of RSL class II genes in *A*. *thaliana*; there was no detectable *AtRSL2*, *AtRSL3*, or *AtRSL5* expression and *AtRSL4* was reduced in the *Atrhd6 Atrsl1* double mutant (Fig 5A) [16]. However, expression of *AtRSL2*, *AtRSL3*, *AtRSL4* and *AtRSL5* was restored in *Atrsl1 Atrhd6* double mutants transformed with *35S*:*BdRSL1* or *35S*:*BdRSL2* gene constructs (Fig 5A). The *35S*:*BdRSL3* transgene partly restored *AtRSL2*, *AtRSL3*, *AtRSL4* expression (Fig 5A). To test if restoration of RSL class II gene expression by *B*. *distachyon* RSL class I genes also restored root hair elongation, we characterized root hair growth in *Atrhd6 Atrsl1* double mutants expressing the *B*. *distachyon* RSL class I genes. While root hairs do not develop on *Atrhd6 Atrsl1* double mutants, root hairs formed on the double mutants transformed with *35S*:*BdRSL1*, *35S*:*BdRSL2* or *35S*:*BdRSL3* gene constructs (Fig 5B); root hair development was indistinguishable from *Atrhd6 Atrsl1 35S*:*RHD6* plants. This indicates that expression of the *BdRSL1*, *BdRSL2* and *BdRSL3* genes can replace the missing RSL class I gene function in the *A*. *thaliana Athd6 Atrsl1* double mutant.

Since RSL class I expression was sufficient for root hair development in *B*. *distachyon*, we tested if RSL class I expression was sufficient for root hair development in *A*. *thaliana*. First, we characterized the pattern of epidermal cell differentiation in *Atrhd6 Atrsl1* double mutants transformed with *35S*:*RHD6* or *35S*:*RSL1* gene constructs. Root hair cells developed in longitudinal cell files and were flanked by files of hairless epidermal cells in the transformed plants—the same pattern observed in wild type—while the *Atrhd6 Atrsl1* mutants were root hairless (Fig 5C). These data indicate that constitutive expression of *A*. *thaliana* RSL class I genes did not induce ectopic root hair cell development in *A*. *thaliana*, unlike *B*. *distachyon* where ectopic RSL class I gene expression resulted in ectopic root hair cell development (Fig 3A).

If the function of RSL class I genes has been conserved since *A*. *thaliana* and *B*. *distachyon* last shared a common ancestor, we predicted that the phenotype of *A*. *thaliana* plants that ectopically express *B*. *distachyon* RSL class I genes would be the same as the phenotype of plants that ectopically express *A*. *thaliana* RSL class I genes. *Atrhd6 Atrsl1* double mutants were transformed with *35S*:*BdRSL1*, *35S*:*BdRSL2* and *35S*:*BdRSL3* transgenes. Files of root hair cells and hairless epidermal cells formed on the *Atrhd6 Atrsl1* double mutants expressing individual RSL class I genes; ectopic hair cells did not develop (Fig 5C). These plants were morphologically indistinguishable from the *Atrhd6 Atrsl1* double mutants transformed with *35S*:*AtRHD6* or *AtRSL1* (Fig 5C and S5B Fig). These data indicate that neither the expression of *B*. *distachyon* nor *A*. *thaliana* RSL class I genes is sufficient for root hair cell development in *A*. *thaliana*. To independently verify that *B*. *distachyon* RSL class I expression was not sufficient for root hair development in *A*. *thaliana* we ectopically overexpressed these genes in wild type plants. Root hair cells developed in longitudinal cell files and were flanked by files of hairless epidermal cells in wild type transformed with *35S*:*BdRSL1*, *35S*:*BdRSL2* or *35S*:*BdRSL3* (S5A and S5B Fig). These data suggest that the pattern of root hair cell differentiation in the *A*. *thaliana* root epidermis cannot be dictated by the spatial pattern of RSL class I gene expression alone.

The different phenotypes that result from the ectopic expression of RSL class I genes in *B*. *distachyon* and *A*. *thaliana* demonstrate that while the pattern of RSL class I gene expression determines the pattern of hair cell differentiation in *B*. *distachyon*, the pattern of expression in *A*. *thaliana* alone cannot account for the pattern of epidermal differentiation.

## Discussion

The function of RSL class I genes as positive regulators of root hair cell development is conserved between *B*. *distachyon* and *A*. *thaliana*. We conclude that RSL class I genes controlled root hair cell development in the common ancestor of grasses and brassicas which existed some time before 200 million years ago [24]. This demonstrates that RSL class I genes are likely to promote root hair cell differentiation in monocots and eudicots even though different spatial patterns of epidermal development can exist among these taxa.

Despite the conservation of RSL class I function between *B*. *distachyon* and *A*. *thaliana*, the different phenotypic consequences of ectopic RSL class I gene expression in these species highlights a major divergence in the mechanism controlling root epidermal development in each. RSL class I expression is sufficient for hair cell development in *B*. *distachyon–*ectopic overexpression of any of the three RSL class I genes induces the development of root hairs in every cell of the root epidermis. This demonstrates that RSL class I proteins can initiate the root hair cell developmental program in the grass. By contrast, ectopic overexpression of AtRSL class I genes in *A*. *thaliana* does not induce the development of ectopic root hair cells in the root epidermis. This suggests that while *A*. *thaliana* RSL class I proteins promote root hair cell development, their expression alone is not sufficient for root hair cell development in other cell types of the root epidermis. These observations could be explained by either (i) *B*. *distachyon* RSL class I genes being functionally different from *A*. *thaliana* RSL class I genes, or (ii) the presence of an activity that represses root hair cell development in *A*. *thaliana* despite the high levels of RSL class I gene activity in the experimental transgenic plants. It was possible to rule out the former because while expression of *B*. *distachyon* RSL class I genes in the *A*. *thaliana Atrhd6 Atrsl1* double mutants was sufficient to restore root hair development in root hair cells, their expression was not sufficient for root hair development in the cells in the hairless epidermal cell position [9]. This suggests that the differences in the ability of RSL class I genes to induce root hair development is due to factors that repress hair development in *A*. *thaliana* in developing hairless epidermal cells that are missing from *B*. *distachyon*. Taken together these data suggest that the mechanism controlling root hair cell differentiation is conserved between *B*. *distachyon* and *A*. *thaliana*, but the mechanism that controls the spatial patterning of the two cells types in the root epidermis is different between the two species.

While RSL class I genes positively regulate root hair cell development in both *B*. *distachyon* and *A*. *thaliana*, their expression patterns differ dramatically. *BdRSL1*, *BdRSL2* and *BdRSL3* are expressed at or soon after the formative asymmetric cell division that forms the future root hair cells. That is, these genes are expressed in the elongations zone and in root hair cells in the differentiation zone, but there is no expression in the meristem. By contrast, *AtRHD6* and *AtRSL1* are expressed in the meristem and no expression of these RSL class I genes is detected in the developing root hair cells in the elongation or differentiation zones [11]. This different timing of RSL class I expression mirrors the morphological differentiation of the two cell types in these species. Epidermal cells become morphologically different relatively late development in the grass—after the last meristematic cell division—while differentiation is morphologically detectable in the meristem of *A*. *thaliana*, where files of relatively short cells are destined to develop root hairs and relatively long cells are destined to become hairless epidermal cells. Therefore, morphological differentiation is first visible in the meristem in *A*. *thaliana* and in the elongation zone of *B*. *distachyon* and this mirrors the patterns of RSL class I gene expression in the two species. Consequently we can reconcile the different expression patterns if RSL class I genes are expressed in future hair cells as soon as they become morphologically different from neighbouring future hairless epidermal cells. Since this event occurs in the meristem of *A*. *thaliana*, RSL class I genes are expressed in the meristem and since this event occurs after the last cell division in *B*. *distachyon*, the genes are in the elongation zone. However this does not explain why the RSL class I genes continue to be expressed during root hair elongation in the grass. It is possible that the continued expression of *BdRSL1*, *BdRSL2* and *BdRSL3* in these cells maintains the growth program active.

We conclude that RSL class I genes positively regulate the development of root hair cells in *B*. *distachyon* which demonstrates that the mechanism of RSL-regulated root hair differentiation has been conserved between Brassicaceae and Poaceae which last shared a common ancestor some time around 200 million years ago [24]. We also present evidence that suggests that the expression patterns RSL class I genes can account for the pattern of root hair cell differentiation found in members of the grass family.

## Materials and Methods

### Plant material and growth

*Brachypodium distachyon* (line Bd21) caryopses (grain) were sterilized in 10% NaClO and 0.1% Triton X-100 with gentle shaking for 15 minutes, then washed with sterilized water 5 times. Grain were then placed on wet filter paper in a Petri dish sealed with cling film and incubated for 2 days in the dark at 5°C (*B*. *distachyon*) to ensure that all grain germinated at the same time. Grain were placed in Petri dishes containing media as in described previously [25] but modified to contain 1% sucrose and 0.5% phytagel (Sigma, UK). Plants were grown vertically in a growth chamber with a 16 hour light period at 23°C for 5 days. *Arabidopsis thaliana* wild type plants of the ecotype Columbia-0 (Col0) were used. *Arabidopsis thaliana* seeds were sterilized in 5% NaClO for 5 min and washed with sterilized water for 5 times. Seeds were sown and germinated in Petri dishes containing sterilized media as in Ma *et al*. [28] modified to contain 1% sucrose and 0.5% phytagel (Sigma, UK). The plants were grown vertically in the growth chamber with a 16 hour light period at 23°C for 5 days.

### Alignment and phylogenetic analysis

Protein sequences were pre-aligned using Multiple Alignment using Fast Fourier Transform (MAFFT) (http://mafft.cbrc.jp/alignment/server/index.html). The bHLH region was then extensively manually aligned in BioEdit (http://www.mbio.ncsu.edu/BioEdit/BioEdit.html). Unambiguous aligned positions were used for the subsequent phylogenetic analyses (S1B Fig). The Jones, Taylor, and Thorton (JTT) model was selected as the best-fitting amino acid substitution model with the Akaike information criterion implemented in ProtTest [26]. A phylogenetic tree was constructed with the aligned bHLH protein sequences using PhyML 3.0 (http://www.atgc-montpellier.fr/phyml/) and thMaximum Likelihood method with the following parameters: SPR &NNI, aLRT SH-like. The constructed tree file was visualized using MEGA [27] (version 6.0; http://www.megasoftware.net/index.html). Logos of amino acid sequence alignments were created with WebLogo (http://weblogo.berkeley.edu/). The multiple alignment to score similarity of amino acid was carried out Mview program (http://www.ebi.ac.uk/Tools/msa/mview/).

Synteny *B*. *distachyon* and *O*. *sativa* genomes were analysed using SyMAP v4.2 software (http://www.symapdb.org/projects/poaceae/), and the ORFs in both *O*. *sativa* and *B*. *distachyon* genome were identified using a BLAST filtering approach (BLASTP, e-value < 0.01). All predicted gene sequences were searched against all available databases of fully sequenced genomes (*O*. *sativa*; http://signal.salk.edu/cgi-bin/RiceGE, *B*. *distachyon*; http://jbrowse.brachypodium.org/JBrowse.html).

### Generation of transgenic plants

*B*. *distachyon* overexpression constructs were based on pVec8- GFP [28] (a gift from Philippe Vain, John Innes Centre, Norwich, UK). The GFP sequences were removed and a unique KpnI site was generated between *ZmUBI* promoter and *Nos* terminator to make the pVec8-overexpression vector. For the constitutive expression of RSL class I genes in *B*. *distachyon*, the coding sequences of RSL class I were amplified from root cDNA with primer pairs that added a KpnI recognition site at both ends: *BdRSL1* F (KpnI): 5’-CGGTACCCTCATGGCAAGCAGGCACG-3’, BdRSL1 R (KpnI): 5’-CGGTACCGCATCATGCATGGCCTAGG-3’ for *BdRSL1* (836bp); BdRSL2 F (KpnI): 5’-CGGTACCCTGATCATGGCATTAGTGCG-3’, *BdRSL2* R (KpnI): 5’-CGGTACCGGTACGTGTTTCCTTCTAGC-3’ for *BdRSL2* (951bp); BdRSL3 F (KpnI): 5’-CGGTACCGCCATGGCTCTAGTGGGTC-3’, *BdRSL3* R (KpnI): 5’-CGGTACCGCTATATACTGCTAGCTCC-3’ for *BdRSL3* (1157bp) (the KpnI site is underlined). The PCR products were subcloned into the pGEM-T Easy Vector (Promega), and sequenced using SP6 and T7 primers to verify the PCR product. Cloned PCR fragments were digested with KpnI and ligated into KpnI-digested pVec8-overexpression plasmid. Sense orientations were confirmed by sequencing with the forward primer 5′-TGATGGCATATGCAGCAGC-3’ from the ZmUBI promoter.

The dsRNAi site of pANDA vector [29], containing attR cassettes at both sites of the GUS linker in the sense and antisense orientations were introduced to pVec8-overexpression KpnI site between *ZmUBI* promoter and *Nos* terminator. RNA silencing constructs carrying the 3’ region of each class I RSL gene were made using the modified pVec8 *dsRNAi* vector to silence the endogenous class I RSL gene. 50 ng cDNA was used for PCR amplification with each of the following gene specific primer sets (underlines are attB site to create entry clones using the BP recombination reaction) BdRSL1 dsRNA F: GGGGACAAGTTTGTACAAAAAAGCAGGCTTCGAATCAATCCTAGGCCATG, BdRSL1 dsRNA R: GGGGACCACTTTGTACAAGAAAGCTGGGTCAGTGACCAGAACTCCGAAC for BdRSL1 dsRNAi (239bp); BdRSL2 dsRNA F: GGGGACAAGTTTGTACAAAAAAGCAGGCTTCGGCAATTCAACTGCTCCAG, *BdRSL2* dsRNA R: GGGGACCACTTTGTACAAGAAAGCTGGGTCCAGGAGTACTACCTACTAC for *BdRSL2* dsRNAi (273bp); *BdRSL3* dsRNA F: GGGGACAAGTTTGTACAAAAAAGCAGGCTTCGCAGAAGCCTCGTCAGTG, *BdRSL3* dsRNA R: GGGGACCACTTTGTACAAGAAAGCTGGGTCAGTTCGGATGGCAAGGTG for *BdRSL3* dsRNAi (207bp) and then PCR product subcloned into the pDONR207 donor vector by a BP clonase reaction (Invitrogen). The final RNA silencing vectors were produced by an LR clonase reaction (Invitrogen) between an entry vector and the modified pVec8 dsRNAi vector. The gateway system (Invitrogen) was used to construct the GFP fusion vector. For the BdRSL class I promoter, the genomic DNA was amplified with each of the following gene specific primer sets (attB site underlined to create entry clones using the BP recombination reaction) and cloned into pDONR207. BdRSL1pro F:GGGGACAAGTTTGTACAAAAAAGCAGGCTTC GTTACTATGGGCTACTGGG, BdRSL1pro R:GGGGACCACTTTGTACAAGAAAGCTGGGTC GAGCTAGGCAGCTATAACAC, for BdRSL1 promoter, BdR2pro F:GGGGACAAGTTTGTACAAAAAAGCAGGCTTC GCAACATGATCGAGGATCC, BdR2pro R:GGGGACCACTTTGTACAAGAAAGCTGGGTC GATCAGTTGATCACGTATCAATC, for BdRSL2 promoter, BdR3pro F:GGGGACAAGTTTGTACAAAAAAGCAGGCTTC CGTATCCGGTGAAAACGAC, BdR3pro R:GGGGACCACTTTGTACAAGAAAGCTGGGTC GGCGGTGGGACTAAGCGG, for BdRSL3 promoter. After sequencing, to confirm the insertion, a GATEWAY LR reaction was then performed with pVEC8-R1-CmR-ccdB-R2:sGFP vector.

Transgenic *B*.*distachyon* plants were generated using *Agrobacterium tumefaciens* mediated transformation method as described previously [33]. Immature embryonic calli were induced from immature embryos cultured on MSB3 + Cu0.6 medium for 6 weeks to produce callus, and then co-cultivated with Agrobacterium strain AGL1 harboring the pVec8 binary vector including target gene, before being transferred to selective medium supplemented with 50 μg/L hygromycin. Hygromycin resistant calli were subsequently regenerated on regeneration media [30]. The transgenic plantlets were then transferred to the greenhouse in pots of John Innes No. 1 compost with a 16-hour light period at 23°C to grow and produce seeds. T3 seeds that were homozygous for the transgene were harvested and several lines tested for gene expression levels and used for further analysis.

For overexpression in *A*. *thaliana*, *BdRSL1*, *BdRSL2*, and *BdRLS3* cDNAs were used and subcloned into the pGEM-T Easy Vector (Promega) (see above). cDNA was inserted into the pCAMBIA1300 binary vector. All binary vectors were transferred into Agrobacterium strain GV3101. *Arabidopsis* plants were transformed using a modified version of the floral dip method [31]. Agrobacterium transformed with a binary vector was grown overnight at 28°C in LB medium with antibiotic (Kanamycin 50 μg/ml and Rifampicin 125 μg/ml) for plasmid selection. 2 ml of this culture was used to inoculate 500 ml of fresh LB media, which contained antibiotics, and incubated for 24 hours at 28°C. Cells were pelleted by centrifugation and then resuspended in an equal volume of infiltration medium (MS salts, 5% (w/v) sucrose, 0.05% (v/v) Silwet L-77 (Vac-in-Stuff, Lehle Seeds)) and 0.15 mM acetosyringone. *Arabidopsis* plants were dipped into the bacterial suspension for 60–90 seconds and covered with a plastic bag for 24 hour to maintain humidity. After removing the bag, plants were returned to normal greenhouse conditions for seed harvesting. Transformants were selected on kanamycin (50 μg/ml).

### Root hair measurements

Images of root hairs of 5 day old seedling were visualized using a Nikon Eclipse E600 epifluorescence light microscope (Nikon, Japan) with Nikon Plan Apo objectives and bright field optics, and a Leica M165 FC stereo microscope and images captured with a Leica DFC310 FX camera. Images were postprocessed with ImageJ (http://rsb.info.nih.gov/ij/) and Adobe Photoshop CS4.

### Gene expression analysis

Total RNA was isolated from *B*. *distachyon* at different times during development. Root RNA was isolated from 5-day-old plants and shoot apical meristem RNA was isolated from 10-day-old-tissues. Total RNA was isolated from 1-month old leaves, shoots and emerging flowers. Root RNA was isolated from *A*. 5-day-old *A*. *thaliana* plants. Total RNA was isolated from frozen plant tissue with the RNeasy Plant Mini Kit (Qiagen) and reverse transcribed using the Super Script III First-Strand Synthesis System for RT-PCR using oligo dT primer (Invitrogen). Semi quantitative RT-PCR was routinely carried out for 30 to 35 cycles, depending on the linear range of PCR amplification for each gene. Each PCR cycle included incubations at 98°C for 10 s, 58°C for 20 s, and 72°C for 30 s. Specific primers were designed to generate RT-PCR products: *BdRSL1* RT: 5’-GAAGAAGCAGTGTGGAGGAAG-3’ and 5’-GACCATGTCAACCTTTGTGCC-3’; *BdRSL2* RT: 5’-GACGTTGCAGGAGATGGTG-3’ and 5’-CGAGCACCTTGACTTGCAG-3’; *BdRSL3* RT: 5’-CAAGCTGCCAAGAGCCGTC-3’ and 5’-CTGTCTCGAGCACCGTGAG-3’; *BdUBI* RT: 5’-CAATGTCAAGGCGAAGATCC-3’ and 5’-GGTCTTCACGAAGATCTGC-3’; *AtRHD6* RT: 5’-CCTAAATCCGCTGGAAACAA-3’ and 5’-CTCTTCGATTCTTGGCTGCT-3’; *AtRSL1* RT: 5’-CCCTAAACTGGCTGGCAATA-3’ and 5’-TCTTGGCTGCTAGGCTTTGT-3’; *AtRSL2* RT: 5’-TCCCCAATGGAACAAAGGTC-3’ and 5’-TCTCGGTGAGCTGAGACCAA-3’; *AtRSL3*: 5’-GGAGCCAGAAATGCGTAGAG-3’ and 5’-GTCTCCACCGTTTGATTCGT-3’; *AtRSL4* RT: 5’-GTGCCAAACGGGACAAAAGT-3’ and 5’-TTGTGATGGAACCCCATGTC-3’; *AtRSL5* RT: 5’-GCAGGAACTTCACGTAATGGA-3’ and 5’-TATACGCTAGGAAACGAAGAGAAA-3’; *EF1α*: RT: 5’-GGTGGTGGCATCCATCTTGTTACA-3’ and 5’-TGAGCACGCTCTTCTTGCTTTCA-3’

### Confocal microscopy

Roots were mounted in 10 ng/ml propidium iodide (Sigma) solution. Confocal images of root epidermal cells were obtained by Leica TCS SP5 confocal laser scanning microscopes using 543-nM and 490-nM laser line for excitation of PI and GFP, respectively. Images were post-processed with ImageJ (http://rsb.info.nih.gov/ij/) and Adobe Photoshop CS4.

### Cryo-scanning electron microscopy

For cryo-scanning electron microscopy, seedlings were placed on moist nitrocellulose paper, mounted on a stub and immersed in liquid nitrogen slush. Roots were transferred to a cold stage. After removal of water by sublimation, roots were sputter coated with gold and observed with a Philips XL30 FEG scanning electron microscope equipped with an Oxford Instruments CT1500 HF cryo-stage at -147°C. Digital images were captured with the maximum pixel density. Image J (http://rsb.info.nih.gov/ij/) and Adobe Photoshop CS4 were used to process the images.

### Whole mount in situ hybridization

In situ hybridization was used to determine the cells in which RSL class I mRNA accumulated. Antisense probes for the class I *RSL* gene were made by amplifying the full coding sequence including the 5’ UTR and 3’ UTR by PCR using full length cDNA primers (see above). PCR fragments were cloned into pGEM-T Easy Vector (Promega). To generate specific antisense probes, full length cDNA was transcribed from the terminal SP6 or T7 RNA polymerase priming sites of these vectors, then treated DNase and precipitated RNA by ethanol. Full length RNA was hydrolyzed by carbonate buffer (200 mM, pH10) to create 150 bp fragments for hybridization. *In vitro* transcription, digoxigenin labeling of RNA probes, tissue preparation, and in situ hybridization were carried out as described in Drea *et al*. [32] and Derbyshire *et al*. [33].

## Supporting Information

S1 Fig RSL class I genes sequences and alignments used in this study. **(A)** RSL sequences from *B*. *distachyon*, *O*. *sativa*, *T*. *aestivum* and *H*. *vulgare* (Fig 1A). The list also includes two genes that were used as outgroups *AtIND* and *OsLAX*. (**B)** Alignment of RSL class I and class II gene sequences used for phylogenetic analysis.
(TIF)

S2 Fig The *BdRSL1* promoter is active in root hair cells. Fluorescence produced from the activity of the *BdRSL1pro*:*sGFP* transgene in root hair cells. Scale bar 50 μm.
(TIF)

S3 Fig Vector constructs used for the overexpression of *BdRSL1*, *BdRSL2*, *BdRSL3*. **(A)** *BdRSL* class I cDNAs (*BdRSL1*, *BdRSL2*, and *BdRSL3*) were inserted between the *ZmUBI* promoter and the *Nos* terminator. Arrows indicate restriction sites and the names of the respective restriction enzymes. **(B)** Root hairs are longer in lines transformed with *ZmUBI*_*pro*_:*BdRSL1*, *ZmUBI*_*pro*_:*BdRSL2* and *ZmUBI*_*pro*_:*BdRSL3* than wild type. Asterisks indicate statistically significant differences between overexpression lines and wild type at **P<0.01 (t-test). Error bar indicates SD.
(TIF)

S4 Fig Steady state mRNA levels are reduced in *Bdrsl1 Bdrsl2*, *and Bdrsl2 Bdrsl3* double knock down lines of *B*. *distachyon*. Steady state levels of *BdRSL1* and *BdRSL2* mRNA are lower in *Bdrsl1 Bdrsl2* double knockdown lines than in wild type. Steady state levels of *BdRSL2* and *BdRSL3* mRNA are lower in *Bdrsl1 Bdrsl2* double knockdown lines than in wild type. Error bar is SD.
(TIF)

S5 Fig Expression of *B*. *distachyon* RSL class I genes in wild type *A*. *thaliana* does not induce ectopic root hair cell development. **(A)** Wild-type (Col-0) *A*. *thaliana* transformed with *35S*:*BdRSL1*, *35S*:*BdRSL2* and *35S*:*BdRSL3*. Scale bar 500 μm. **(B)** Mean root hair number mm^-1^ (± SD) in lines transformed with *35S*:*BdRSL1*, *35S*:*BdRSL2* and *35S*:*BdRSL3* is indistinguishable from wild-type.
(TIF)

S1 Table Percentage amino acid identity between the bHLH and RSL domain of *A*. *thaliana* and *B*. *distachyon* RSL class I proteins. (PDF)
