# Correction: ROOT HAIR DEFECTIVE SIX-LIKE Class I Genes Promote Root Hair Development in the Grass *Brachypodium distachyon*

**DOI:** 10.1371/journal.pgen.1006480

**Published:** 2016-12-02

**Authors:** Chul Min Kim, Liam Dolan

There is an error in Fig 5C. The image for the *Atrhd6 Atrsl1 35*:*BdRSL1* root is duplicated and appears as the image for the *Atrhd6 Atrsl1 35*:*AtRSL1* root. Please view the correct Fig 5 here, with the correct image for the *Atrhd6 Atrsl1 35*:*AtRSL1* root.

**Fig 5:**
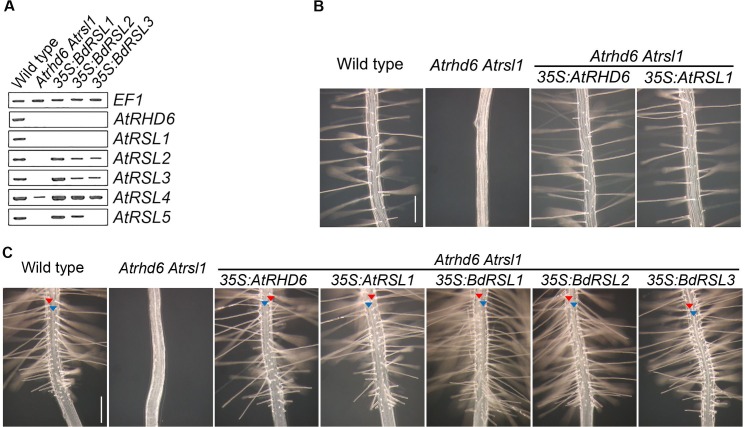
The function *B*. *distachyon* and *A*. *thaliana* RSL class I genes is conserved. (A) Steady state levels of AtRSL class I and class II mRNA in *Atrhd6 Atrsl1* double mutants, and in *Atrhd6 Atrsl1* double mutants transformed BdRSL class I genes under the control of the *35S* promoter. Lane 1: wild type, Lane2: *Atrhd6 Atrsl1* double mutant, Lane3: *Atrhd6 Atrsl1 35S*:*BdRSL1*, Lane4: *Atrhd6 Atrsl1 35S*:*BdRSL2*, Lane5: *Atrhd6 Atrsl1 35S*:*BdRSL3*. (B) Phenotype of wild type; *Atrhd6 Atrsl1* double mutant; *Atrhd6 Atrsl1 35S*:*AtRHD6; Atrhd6 Atrsl1 35S*:*AtRSL1*. Scale bar 200 μm. (C) Files of root hair cells and hairless epidermal cells form in wild-type, *Atrhd6 Atrsl1 35S*:*AtRHD6*, *Atrhd6 Atrsl1 35S*:*AtRSL1*, *Atrhd6 Atrsl1 35S*:*BdRSL1*, *Atrhd6 Atrsl1 35S*:*BdRSL2* and *Atrhd6 Atrsl1 35S*:*BdRSL3*. *Atrhd6 Atrsl1* double mutants do not develop root hairs. Red arrowhead indicates a hair cell file, blue arrowhead indicates the position of a hairless epidermal cell file. Scale bar 200 μm.

## References

[1] Kim CM, Dolan L (2016) ROOT HAIR DEFECTIVE SIX-LIKE Class I Genes Promote Root Hair Development in the Grass *Brachypodium distachyon*. PLoS Genet 12(8): e1006211 doi:10.1371/journal.pgen.1006211 27494519 10.1371/journal.pgen.1006211 PMC4975483

