# Supplementary figures and images for: ROOT HAIR DEFECTIVE SIX-LIKE Class I Genes Promote Root Hair Development in the Grass Brachypodium distachyon
Source: PLoS Genet. 2016 Aug 5;12(8):e1006211. doi: 10.1371/journal.pgen.1006211 (PMC4975483; doi:10.1371/journal.pgen.1006211)

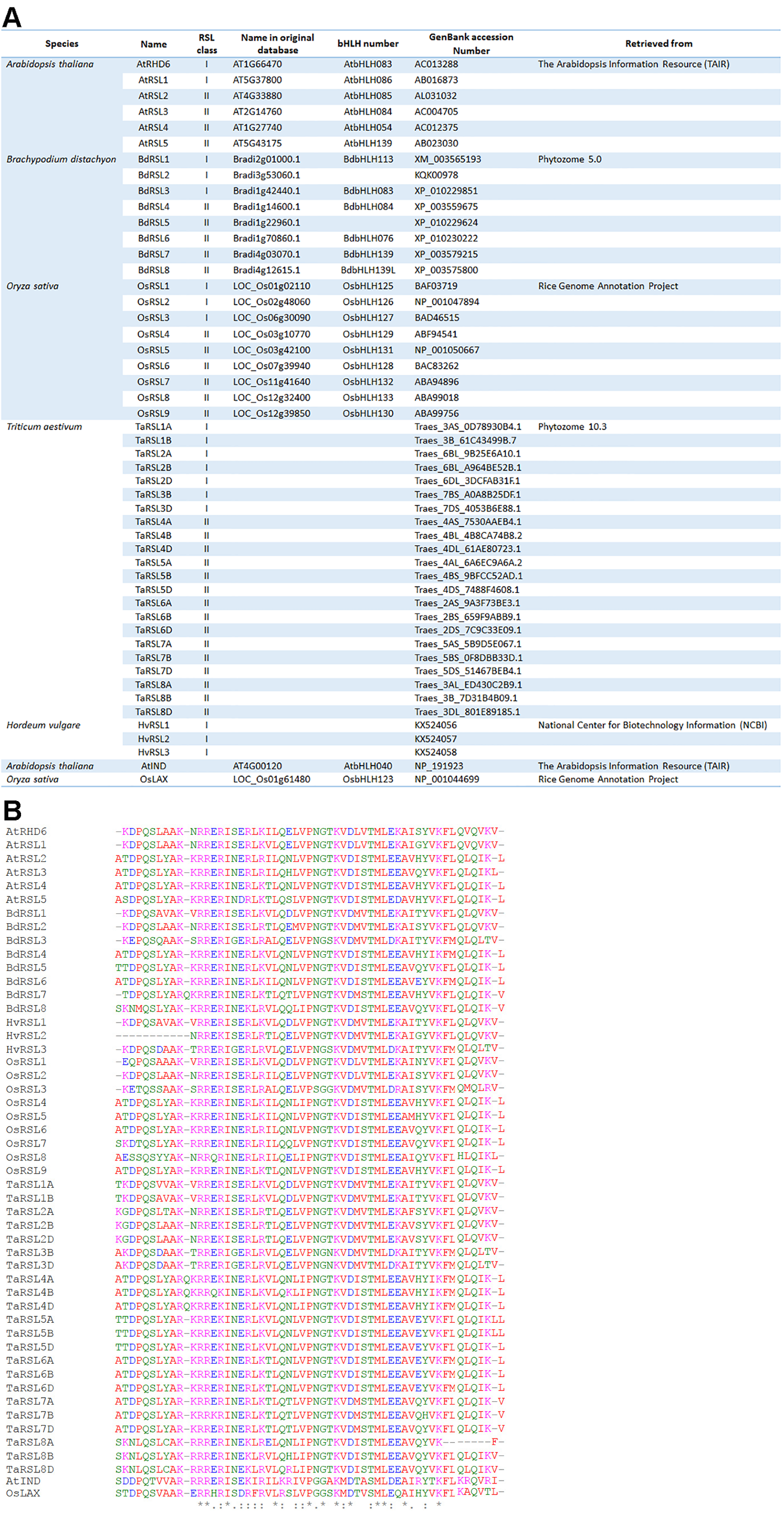

Supplement: S1 Fig — (A) RSL sequences from B. distachyon, O. sativa, T. aestivum and H. vulgare (Fig 1A). The list also includes two genes that were used as outgroups AtIND and OsLAX. (B) Alignment of RSL class I and class II gene sequences used for phylogenetic analysis. (TIF) [file pgen.1006211.s001.tif]

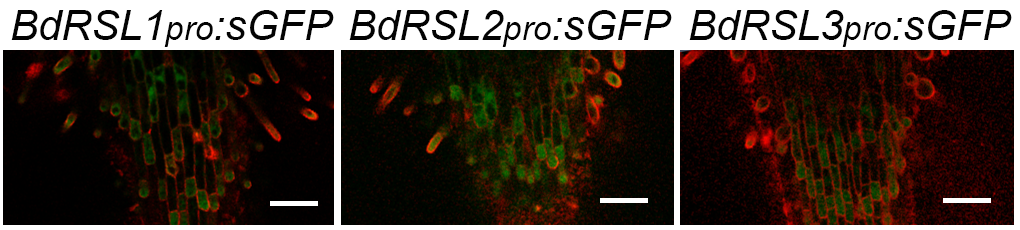

Supplement: S2 Fig — Fluorescence produced from the activity of the BdRSL1pro:sGFP transgene in root hair cells. Scale bar 50 μm. (TIF) [file pgen.1006211.s002.tif]

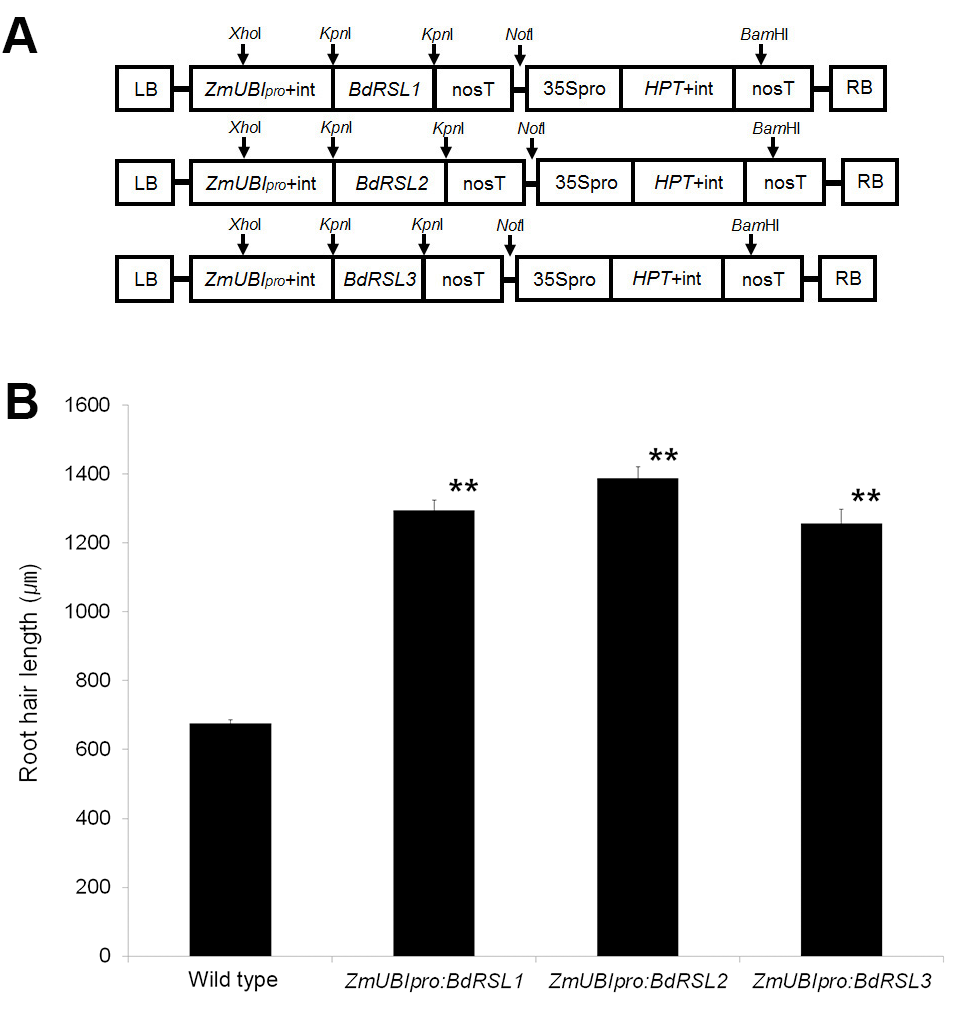

Supplement: S3 Fig — (A) BdRSL class I cDNAs (BdRSL1, BdRSL2, and BdRSL3) were inserted between the ZmUBI promoter and the Nos terminator. Arrows indicate restriction sites and the names of the respective restriction enzymes. (B) Root hairs are longer in lines transformed with ZmUBIpro:BdRSL1, ZmUBIpro:BdRSL2 and ZmUBIpro:BdRSL3 than wild type. Asterisks indicate statistically significant differences between overexpression lines and wild type at **P<0.01 (t-test). Error bar indicates SD. (TIF) [file pgen.1006211.s003.tif]

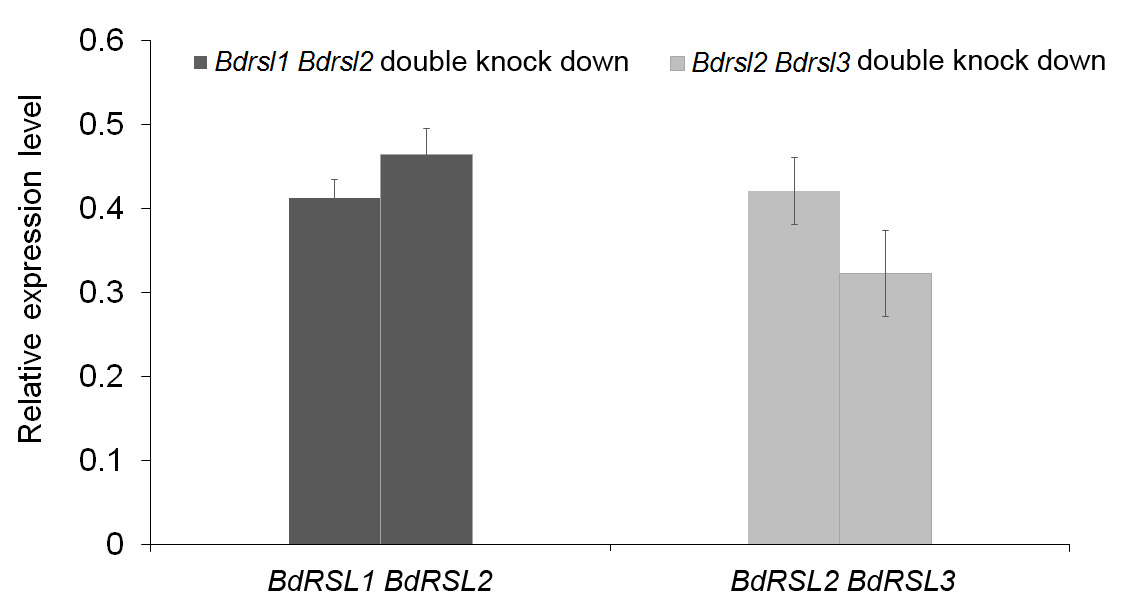

Supplement: S4 Fig — Steady state levels of BdRSL1 and BdRSL2 mRNA are lower in Bdrsl1 Bdrsl2 double knockdown lines than in wild type. Steady state levels of BdRSL2 and BdRSL3 mRNA are lower in Bdrsl1 Bdrsl2 double knockdown lines than in wild type. Error bar is SD. (TIF) [file pgen.1006211.s004.tif]

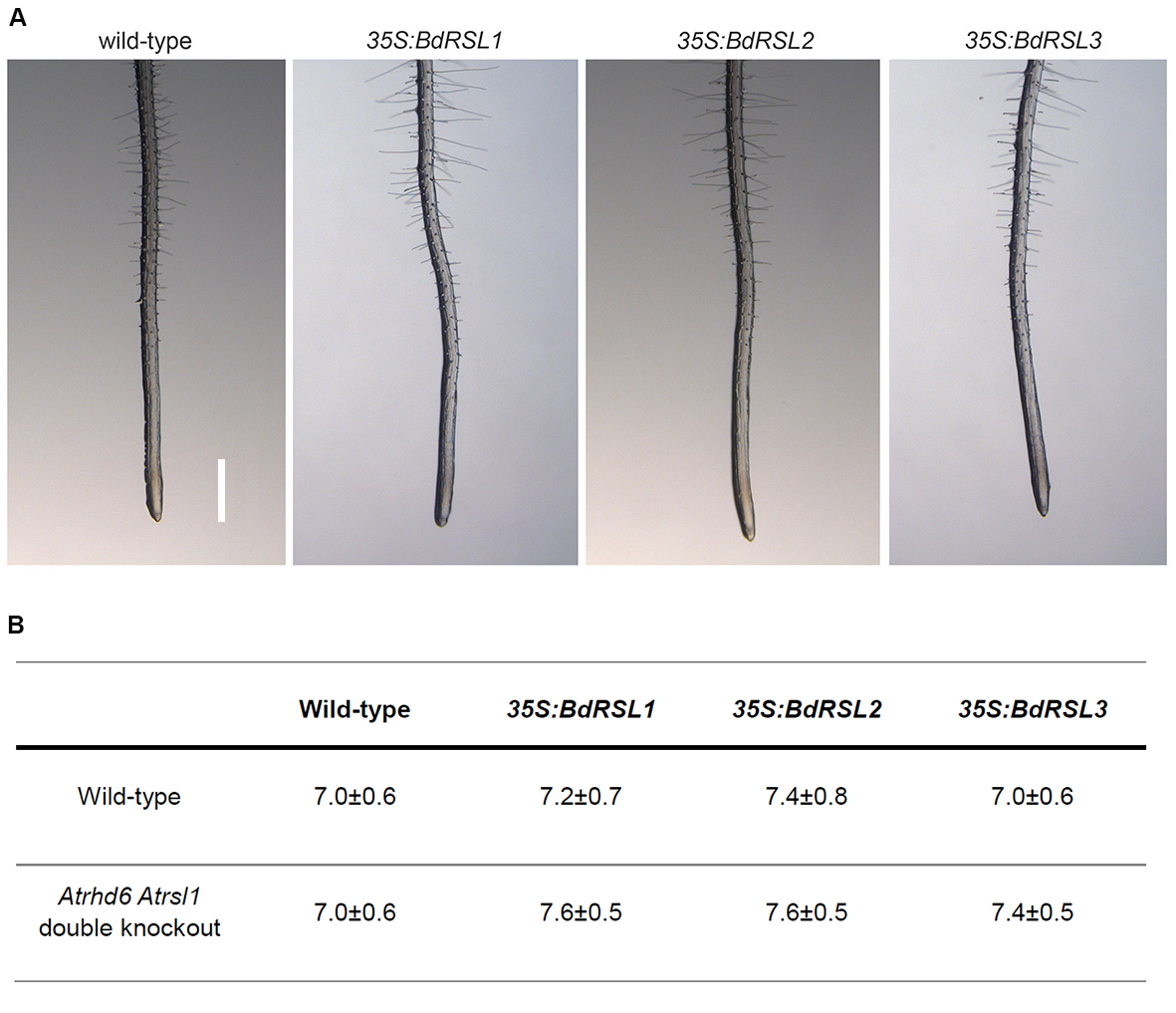

Supplement: S5 Fig — (A) Wild-type (Col-0) A. thaliana transformed with 35S:BdRSL1, 35S:BdRSL2 and 35S:BdRSL3. Scale bar 500 μm. (B) Mean root hair number mm-1 (± SD) in lines transformed with 35S:BdRSL1, 35S:BdRSL2 and 35S:BdRSL3 is indistinguishable from wild-type. (TIF) [file pgen.1006211.s005.tif]
