# Supplementary material for: ROOT HAIR DEFECTIVE SIX-LIKE Class I Genes Promote Root Hair Development in the Grass Brachypodium distachyon
Source: PLoS Genet. 2016 Aug 5;12(8):e1006211. doi: 10.1371/journal.pgen.1006211 (PMC4975483; doi:10.1371/journal.pgen.1006211)
Supplement: S1 Table — (PDF) [file pgen.1006211.s006.pdf]

	AtRHD6	AtRSL1
**AtRHD6**	100.0	
**AtRSL1**	93.7	100.0
**BdRSL1**	84.1	84.1
**BdRSL2**	88.9	87.3
**BdRSL3**	73.0	71.4
